# The Biological Functions and Clinical Values of Exosomal Circular RNAs in Hepatocellular Carcinoma

**DOI:** 10.3389/fonc.2022.885214

**Published:** 2022-04-21

**Authors:** Ying Zhao, Jinmei Yao

**Affiliations:** Department of Clinical Laboratory, The First Affiliated Hospital, College of Medicine, Zhejiang University; Key Laboratory of Clinical In Vitro Diagnostic Techniques of Zhejiang Province, Hangzhou, China

**Keywords:** hepatocellular carcinoma, exosome, circRNAs, metastasis, prognosis

## Abstract

Hepatocellular carcinoma (HCC) exacts a heavy disease burden and is currently the second most common cause of cancer-related deaths worldwide. HCC usually lacks obvious symptoms in the early stage, and most HCC patients are diagnosed at advanced stages with poor prognosis. Circular RNAs (circRNAs) are single-stranded RNAs that form covalently closed loops and are stable in exosomes. Exosomes are known as important messengers of the cross-talk between tumor and immune cells. Accumulating studies have demonstrated the promoter or suppressor roles of exosomal circRNAs in the carcinogenesis, progression, and metastasis of HCC. In this review, we summarized the current studies on the biological functions and diagnostic and prognostic values of exosomal circRNAs in HCC progression.

## Introduction

Hepatocellular carcinoma (HCC) is the primary malignancy of hepatocytes. Active hepatitis C and B continue to drive most of the global burden of HCC (1, 2). Early-stage HCC can be treated curatively by surgical resection, local ablation, or liver transplantation, and early diagnosis could have a good prognosis with a 5-year survival rate of more than 70% (3, 4). However, HCC usually lacks obvious symptoms in the early stage, and more than 75% of HCC patients are diagnosed at the advanced stage when the tumor is unresectable, making the 5-year survival rate of patients with HCC less than 16% (1, 3). Early detection of HCC is always based on monitoring with ultrasonography and a number of serological markers, namely, alpha-fetoprotein (AFP), Lens culinaris agglutinin-reactive AFP (AFP-L3), Glypican-3, and osteopontin, but their diagnostic accuracies have been shown to be insufficient (5). Therefore, there is an urgent need to explore novel biomarkers for HCC diagnosis.

Circular RNAs (circRNAs) are single-stranded RNAs that form covalently closed loops (5). Recently, circRNAs have been recognized as key factors in tumor development and have been found to be abundant and stable in exosomes (6). Exosomes are known as important messengers of the cross-talk between tumor and immune cells (7, 8). CircRNAs may exert their functions *via* exosomes and tumor stem cells (9). CircRNAs in exosomes provide necessary energy for tumor growth, participate in mutation metabolism in tumors and regulate signal pathways by transporting non-coding RNAs (10). Some evidence indicates that exosomal CircRNAs may contribute to HCC cell proliferation, migration, invasion, and glycolysis by regulating their targeted microRNAs and downstream tumor-related signaling pathways in HCC (11, 12). In this review, we summarized the current studies on the origin, biological functions, and diagnostic and prognostic value of exosomal circRNAs in HCC progression.

## Biogenesis and Function of Exosome

Exosomes are membrane-bound extracellular vesicles (EVs) that originate from the limiting membrane of late endosomes. Exosomes are small, single-membrane, secreted organelles with an average diameter of about 100 nm (13–16). Additionally, exosomes play significant roles in various biological functions, namely,intra- and inter-cellular communication in both physiological and pathological contexts, and the transfer of biomolecules such as proteins, enzymes, lipids, and RNAs in various diseases (17–19). The biogenesis of exosomes begins with the endosome system. After various maturation processes, the nuclear endosomal membrane invaginates to form multivesicular bodies (MVBs). In addition to leading to the generation of intraluminal vesicles (ILVs), MVBs can also fuse with lysosomes for degradation (20). The biogenesis and secretion of exosomes appear to involve several mechanisms. ESCRT-mediated MVB biogenesis is the most extensively described pathway, and it depends on cell type or intracellular homeostasis (20, 21). Some studies pointed out that exosomes were considered promising biomarkers for the diagnosis, treatment, and prognosis of various diseases, especially those that played a key role in the establishment of tumor microenvironment, tumor progression, invasion, metastasis, chemoresistance, and targeted drug delivery (14, 22–26).

## Origin and Function of Exosomal circRNAs

CircRNAs are a new class of endogenous noncoding RNAs with covalently closed loop structures that lack 5′ caps, 3′ polytails, and polyadenylated tails (27). CircRNAs are evolutionarily conserved, show cell-specific expression patterns, and regulate themselves independently of their linear transcripts (28). The study by Li et al. (29) first reported the existence of abundant exosomal circRNAs, which represented a novel class of stable RNA species in exosomes by RNA-seq analyses. Some studies have discovered that circRNAs are identified in cellular RNAs and can be transferred to exosomes, and subsequently the molecular information is transferred to recipient cells (27, 30).

Exosomes are important mediators of intercellular communication, particularly in the tumor microenvironment (31). Some evidence demonstrated that circRNAs had abundant miRNA binding sites and exerted important biological functions by stabilizing microRNAs (miRNAs), regulating alternative splicing and acting as miRNA inhibitors (‘sponges’), protein ‘decoys’, or by encoding small peptides (31, 32). Tumor cell-derived exosomal circRNAs can act on target cells or organs by the transport of exosomes and play oncogenic or tumor suppressive roles during tumor development (33). With the rise of exosome research, some researchers have investigated the relationship between exosomes and circRNAs in tumors and concluded that circRNAs in exosomes can work as novel biomarkers for tumor diagnosis, thus providing a new development direction for tumor diagnosis (33, 34). Dou et al. (30) found that circRNAs had been detected in cancer-derived exosomes in higher abundance than mutant KRAS cells and suggested a potential involvement of circRNAs in oncogenesis. This finding implied that exosomal circRNAs had the potential as tumor biomarkers. The study by Shang et al. (35) found that circPACRGL was significantly upregulated in colorectal cancer cells (CRC) after tumor-derived exosomes addition and indicated that cancer-derived exosomal circPACRGL played an oncogenic role in CRC proliferation and metastasis. In gastric cancer tissues and serum, the expression of exosomal circSHKBP1 was significantly increased and related to advanced TNM stages and poor prognosis, while exosomal circSHKBP1 regulated the miR-582-3p/HUR/VEGF pathway, suppressed HSP90 degradation, and promoted GC cell proliferation, migration, invasion, and angiogenesis (36). Hsa_circ_0074854 can be transferred from HCC cells to macrophages *via* exosomes, and the expression of Hsa_circ_0074854 was upregulated. Downregulation of hsa_circ_0074854 can suppress HCC migration and invasion by interacting with HuR and suppressing macrophage M2 polarization (8). Mesenchymal stem cell (MSC)-derived exosomal circular RNAs were a promising treatment for disease. The MSC-derived exosomal circFBXW7 inhibited the proliferation, migration, and invasion of synovial cells and the inflammatory response in rheumatoid arthritis *via* sponging miR-216a-3p and releasing HDAC4 (37). The study by Preußer et al. (38) found circRNAs were particularly abundant in human platelets compared with other hematopoietic cell types and were packaged and released in both microvesicles and exosomes derived from platelets. Since platelets are associated with hemostasis, inflammation, and cancer metastasis, studies on exosomal circRNAs may provide a novel avenue for many disease diagnoses and therapies (38).

## The Biological Functions of Exosomal circRNAs in HCC

**Figure 1:**
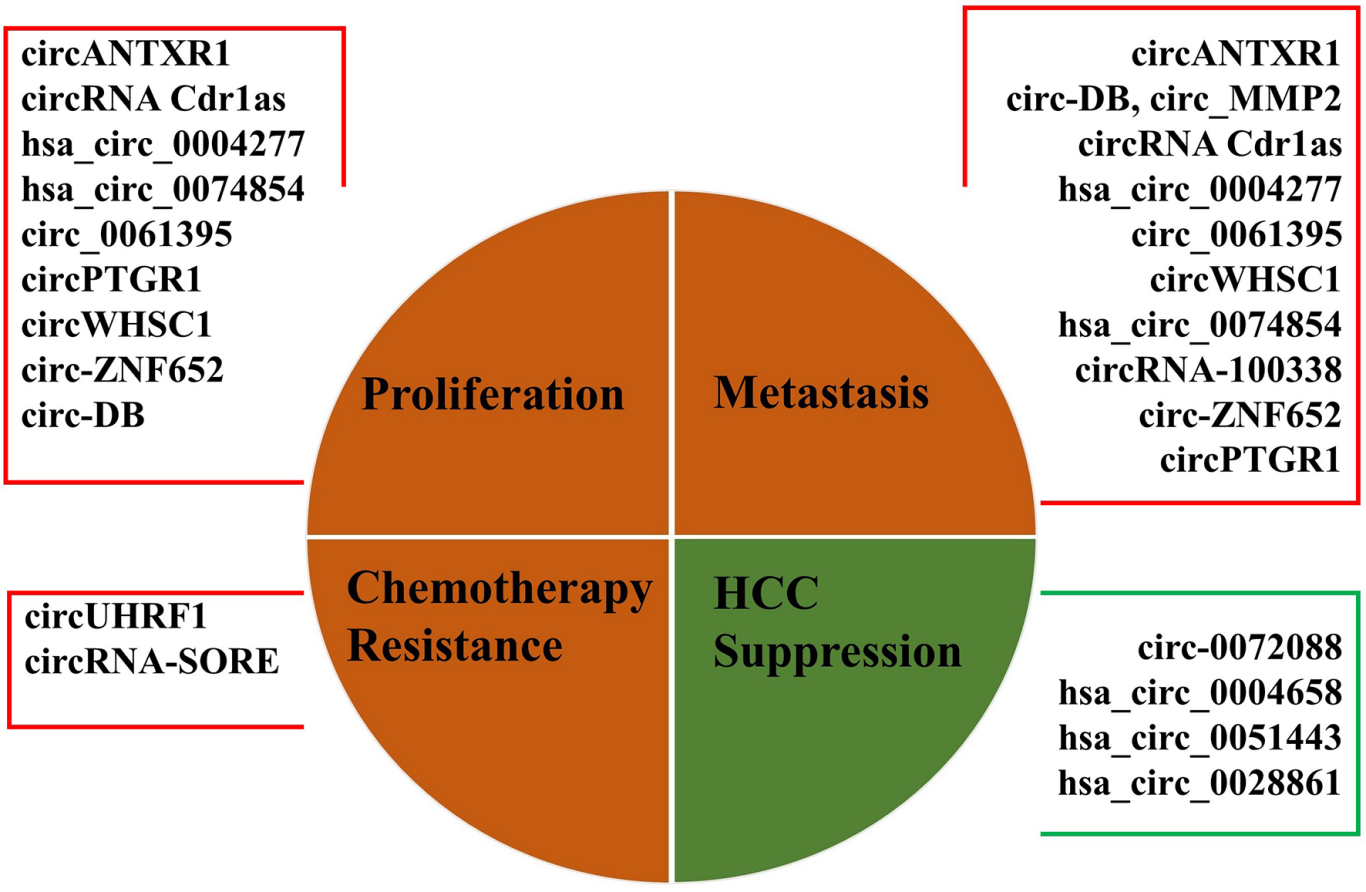
Representation of main biological functions of dysregulated exosomal circRNAs in HCC.

While the exact biological function of most circRNAs in HCC is still largely unknown, the abnormal expressions of exosomal circRNAs were found in tissues, body fluids, and serum/plasma of patients with HCC (39). We summarized the current studies to identify the relationship between exosomal circRNAs and HCC more clearly (shown in **Table 1** and **Figure 1**). Recently, abundant bodies of evidence have revealed the relationship between exosomal circRNAs and HCC progression, namely, proliferation, apoptosis, invasion, metastasis, and glycolysis of HCC cells, resistance mechanisms in HCC therapy, epithelial to mesenchymal transition (EMT) of HCC cells, angiogenesis, recurrence, and mortality, by regulating their targeted miRNAs and downstream tumor-related signaling pathways (41, 46, 47, 51, 55, 59). In **Table 1**, most of the upregulated circRNAs are positively associated with HCC progression, except for exosomal circ-0072088 and hsa_circ_0004658. Furthermore, hsa_circ_0051443 and hsa_circ_0028861 are downregulated and inhibit HCC progression.

**Table 1:** The biological functions and potential clinical values of exosomal circular RNAs in HCC.

CircRNAs	Expression	Parent cell	Target cell	Pathway	Potential clinical value	Biological function	Ref.
Circ-0072088	up	Huh-7	Hep3B	miR-375/MMP-16	Diagnosis and prognosis	inhibit the metastasis of HCC cells	Lin et al. (40)
CircUHRF1	up	HCC cells	NK cells	TIM-3/miR-449c-5p	Therapeutic strategy	inhibit NK cell function; drive resistance to anti-PD1 immunotherapy	Zhang et al. (41)
CircANTXR1	up	HuH-7	HCCLM3	miR-532-5p/XRCC5	Diagnostic biomarker and therapeutic target	facilitate HCC proliferation and metastasis	Huang et al. (42)
CircRNA Cdr1as	up	HepG2/SMMC-7721	293T cells	miR-1270/AFP	Therapeutic target	promote the progression of HCC	Su et al. (43)
CircRNA-SORE (circRNA_104797 or circ_0087293)	up	sorafenib-resistant HCC cells	HCC cells	PRP19/YBX1	Sorafenib resistance monitoring	sorafenib resistance in HCC	Xu et al. (44)
circTMEM45A (hsa_circ_0066659)	up	MHCC97H cells	Hep3B cells	miR-665/IGF2	Diagnostic biomarker and therapeutic target	promote HCC progression	Zhang et al. (45)
hsa_circ-0004277	up	HepG2 and SMMC-7721 cells	HL-7702 cells	ZO-1/EMT	Therapeutic target	promote the proliferation and migration of HCC cells	Zhu et al. (46)
hsa_circ_0061395	up	–	–	miR-877-5p/PIK3R3	New perspective	facilitate HCC progression	Yu et al. (47)
CircWHSC1	up	–	–	HOXA1/miR-142-3p	Diagnostic marker	facilitate HCC cell growth and metastasis	Lyu et al. (48)
CircWDR25 (hsa-circRNA-0004310)	up	Hep3B, SMMC-7721, HCCLM3	LX-2	–	Prognostic marker	Participate in the occurrence and development of HCC	Liu et al. (49)
hsa_circ_0070396	up	–	–	–	Diagnostic biomarker	participate the progress of HCC	Lyu et al. (50)
hsa_circ_0074854	up	HepG2	macrophages	HuR/macrophage M2 polarization	Diagnostic and therapeutic marker	enhance migration, invasion and EMT of HCC cells	Wang et al. (8)
circFBLIM1	up	–	SNU-387 and Huh7 cells	miR-338/LRP6	Therapeutic target	promote HCC progression and glycolysis	Lai et al. (51)
circRNA-100338	up	Hep3B and MHCC97H cells	HUVEC	proangiogenic activity	Risk indicator	enhance angiogenesis and HCC metastasis	Huang et al. (52)
Circ-ZNF652	up	HCC cells	SNU-387 and Huh7 cells	miR-29a-3p/GUCD1	Therapeutic target	contribute to HCC cell proliferation, migration, invasion and glycolysis	Li et al. (11)
hsa_circ_0004658	up	macrophages	SMMC-7721 and HepG2	miR-499b-5p/JAM3	Diagnostic biomarker and therapeutic target	inhibit tumor progression and promote apoptosis in HCC cells	Zhang et al. (53)
Circ-DB	up	adipocytes	HCC cells	miR-34a/USP7/Cyclin A2	Risk indicator	promotes HCC growth and reduces DNA damage	Zhang et al. (6)
Circ_MMP2 (has-circ_0039411)	up	97H or LM3 cell	L02 and HepG2	MMP2/miR-136-5p	Therapeutic target	promotes HCC metastasis	Liu et al. (54)
circAKT3	up	–	–	–	Prognostic marker	associate with a higher risk of HCC recurrence and mortality	Luo et al. (55)
DECs (hsa_circ_0004001 hsa_circ_0004123 hsa_circ_0075792)	up	–	–	targeting miRNAs/VEGF/VEGFR, PI3K/Akt, etc.	Diagnostic biomarker	affect the occurrence and progression of HCC	Sun et al. (56)
CircPTGR1 (hsa_circ_0008043 hsa_circ_0003731 hsa_circ_0088030)	up	LM3 cell	HepG2, and 97 L cells	miR449a–MET	Prognostic biomarker and therapeutic target	enhance the potential of cell migration and invasion	Wang et al. (57)
hsa_circ_0051443	down	normal cells (HL-7702)	HCC cells (HuH7 and Hep3b)	BAK1/miR-331-3p	Predictor and therapeutic target	suppress HCC progression	Chen et al. (58)
hsa_circ_0028861	down	–	–	miR-1254, miR-3141-5p/integrin, VEGF, PI3K/Akt, mTOR, etc.	Diagnostic Biomarker	influence HCC progression and downstream tumor-related signaling pathways	Wang et al. (12)

‐, not provided.

Some exosome circRNAs can act on target cells or organs through the transport of exosomes, and then participate in the regulation of proliferation, apoptosis, invasion, metastasis, and glycolysis of HCC cells. Hsa_circ_0004658 secreted by RBPJ overexpressed macrophages can inhibit proliferation and induce apoptosis in HCC cells by sponging miR-499b-5p and downregulating JAM3 expression (53). Circ_0061395 was upregulated in serum exosomes of HCC patients. Circ_0061395 inhibition reduced malignant behavior of HCC cells, induced cell cycle arrest, apoptosis, repressed proliferation, invasion, and migration of HCC cells, by regulating the miR-877-5p/PIK3R3 axis (47). Circ-ZNF652 was upregulated in the exosomes derived from HCC patients and HCC cells. Circ-ZNF652 was a sponge of miR-29a-3p, and GUCD1 was a target gene of miR-29a-3p. Therefore, circ-ZNF652 contributed to HCC cell proliferation, migration, invasion, and glycolysis by regulating the miR-29a-3p/GUCD1 axis (11). Circumstances were also involved in the transport of circANTXR1 in HCC cells. Huang et al. (42) found that MiR-532-5p could be sponged by circANTXR1 and XRCC5 was a target of miR-532-5p. They indicated circANTXR1 silencing can inhibit HCC cell progression *in vitro* and suppress HCC tumor growth *in vivo* through the miR-532-5p/XRCC5 axis. CircTMEM45A and CircWHSC1 expressions were upregulated in HCC tissues and cells. CircWHSC1 played a tumor-promoting role in HCC by elevating HOXA1 through sponging miR-142-3p, and circTMEM45A acted as a miR65 sponge to relieve the repressive effect of miR-665 on its target insulin growth factor 2(IGF2), upregulation of IGF2 and HCC progression (45, 48). Additionally, circ0051443 was mainly packaged in exosomes and significantly lower in the plasma and tissues of patients with HCC compared with healthy controls. Circ-0051443 was transmitted from normal cells to HCC cells *via* exosomes and suppressed the malignant biological behavior by promoting cell apoptosis and arresting the cell cycle (58).

Exosomes produced by tumor cells play a role in epithelial–mesenchymal transition (EMT) and tumor metastasis (60). Widespread metastases remain a major challenge for therapy and prognosis of HCC (2). EMT is an important biological process that is closely associated with cell migration and invasion (11). Increasing evidence indicates that exosomal circRNAs derived from tumor cells participate in EMT and tumor metastasis and provide a new mechanism for the interaction between liver cancer metastasis and angiogenesis (46, 52). Huang et al. (52) first found the overexpression or knockdown of exosomal circRNA-100338 significantly enhanced or reduced the proliferation, angiogenesis, permeability, and metastasis of HCC cells. Additionally, they also observed that sustained high expression of exosomal circrna-100338 in the serum of HCC patients undergoing radical hepatectomy may be a risk indicator of lung metastasis and poor survival rate. In the study by Zhu et al. (46), circ-0004277 was significantly upregulated in HCC cells, tissues, and plasma exosomes, while circ-0004277 overexpression significantly induced EMT-related transcription factor ZEB-1 upregulated and ZO1 downregulated. Then they suggested the overexpression of circ-0004277 enhanced the proliferation, migration, and EMT of HCC cells *in vivo* and *in vitro* by inhibiting ZO-1 and promoting EMT progression (46). The findings by Liu et al. (54) suggest that cell migration, invasion, and EMT progress were promoted after circ_MMP2 was delivered by 97H- or LM3-secreted exosomes into L02 and HepG2 cells by sponging miR-136-5p to enhance MMP2 expression.

Since HCC is a highly heterogeneous cancer, patients with HCC show varying sensitivity to treatment options (60). Additionally, the high recurrence rate of HCC leads to poor prognosis. There is an urgent need for new prognostic biomarkers to help identify drug resistance and recurrence. Zhang et al. (41) reported that exosomal circUHRF1 was predominantly secreted by HCC cells, circUHRF1 inhibited NK cell function by upregulating the expression of TIM-3 *via* degradation of miR-449c-5p, thereby promoting immune evasion and resistance to anti-PD1 immunotherapy in HCC. Sorafenib has shown survival benefits for individuals with advanced HCC, suggesting that molecular-targeted therapies could be effective in this chemoresistant cancer (61). However, sorafenib resistance significantly limits its therapeutic efficacy. Xu et al. (44) found that circRNA-SORE was upregulated in sorafenib-resistant HCC cells and transported by exosomes to spread sorafenib resistance among HCC cells. CircRNA-SORE bonded with the oncogenic protein YBX1 and blocked PRP19-mediated YBX1 degradation. They suggested that sorafenib resistance could be overcome by targeting circRNA-SORE or YBX1. Luo et al. (55) found the expression of exosomal circAKT3 in HCC patients was significantly increased compared with healthy subjects, and HCC patients with high exosomal circAKT3 had higher tumor recurrence rates and higher mortality.

## Relationships Between Exosomal circRNAs and Clinicopathological Characteristics in HCC

In **Table 2**, we summarized the relationships between exosomal circRNA expression and clinicopathological characteristics of HCC patients. Zhang et al. (41) explored the relationship between circUHRF1 expression and the clinicopathological characteristics of 240 HCC patients, and their results showed that HCC patients with circUHRF1 high expression had a larger tumor size and more microvascular invasion than those with circUHRF1 low expression. The study by Lyu et al. (50) showed that a higher expression level of exosomal circ_0070396 was closely related to tumor size and liver encapsulation invasion. However, the exosomal circRNA-SORE and has_circ_0028861 had no correlation with tumor size, tumor node metastasis (TNM), lymph node metastasis (LNM), vascular invasion, and extrahepatic metastasis (12, 44). Huang et al. (52) found that the high expression rate of circRNA-100338 in the serum of HCC patients at three weeks post-operation was closely associated with TNM stages, vascular invasion, extrahepatic metastasis, and satellite foci, but not with gender and age. In another study, the expressions of exosomal circ-0072088, circANTXR1, circTMEM45A, hsa_circ_0061395, and hsa_circ_0004001 were significantly upregulated in patients with HCC and positively correlated with TNM stages and tumor size but not with gender or age. In addition, the expression of circTMEM45A was also related to vascular invasion (40, 42, 45, 47, 56). Furthermore, high levels of hsa_circ_0004123 and hsa_circ_0075792 expression were positively correlated with tumor size and the TNM stages, but not with age, gender, and LNM (56). These existing bodies of evidence stated that TNM stage, tumor size, and vascular invasion were most closely related to the abnormal expressions of exosomal cirRNAs, but age, gender, number of tumors, and LNM showed no significant relationship.

**Table 2:** Relationship between exosomal circRNAs and clinicopathologic characteristics in HCC.

circRNAs	Expression	N(L/H)	Gender	Age	TS	TNM	LNM	NT	LC	VI	EM	SF	Ref.
circ-0072088	up	25/25	N	N	Y	Y	–	–	N	–	–	–	Lin et al. (40)
circUHRF1	up	120/120	N	N	Y	N	–	N	N	Y	–	–	Zhang et al. (41)
circANTXR1	up	35/35	N	N	Y	Y	–		–	–	–	–	Huang et al. (42)
circRNA-SORE	up	30/30	N	N	N	–	N	N	N	N	N	N	Xu et al. (44)
circTMEM45A	up	34/34	N	N	Y	Y		N	N	Y	–	–	Zhang et al. (45)
hsa_circ_0061395	up	12/18	N	N	Y	Y	N	–	–	N	–	–	Yu et al. (47)
hsa_circ_0070396	up	42/69	N	N	Y	–	–	N	–	N	–	–	Lyu et al. (50)
circRNA-100338	up	23/16	N	N	–	Y	–	–	N	Y	Y	Y	Huang et al. (52)
hsa_circ_0028861	down	56	N	N	N		N	–	–	N	N	–	Wang et al. (12)
hsa_circ_0004001	up	35/36	N	N	Y	Y	N	–	–	–	–	–	Sun et al. (56)
hsa_circ_0004123	up	36/35	N	N	Y	N	N	–	–	–	–	–	Sun et al. (56)
hsa_circ_0075792	up	40/31	N	N	N	Y	N	–	–	–	–	–	Sun et al. (56)

Y, correlation; N, no correlation; Number of patients; L/H: low expression/high expression; TS, Tumor size; TNM, Tumor Node Metastasis; LNM, Lymph node metastasis; NT, Number of tumors; LC, Liver cirrhosis; VI, vascular invasion; EM, Extrahepatic metastasis; SF, Satellite foci; ‐, not provided.

## The Diagnostic and Prognostic Values of Exosomal circRNAs in HCC

Despite advances in medical, locoregional, and surgical therapies, HCC remains to be high mortality due to the recurrence and metastasis after surgical resection (61). HCC derived exosomes could redirect metastasis of tumor cells which lack the ability to metastasize to a specific organ (62). Exosomes can shuttle circRNAs between cells, and regulate cell differentiation and tissue development (63). Recently, some studies indicated the exosomal circRNAs could serve as biomarkers for the diagnosis and prognosis of HCC (12, 41, 46, 49, 50). We summarized recent studies on exosomal circRNAs as diagnostic and prognostic biomarkers of HCC (shown in **Tables 3**, **4**).

**Table 3:** Exosomal circRNAs serve as potential diagnostic biomarkers for HCC.

CircRNAs	ROC	95 CI%	Sensitivity (%)	Specificity (%)	Ref.
circ-0072088	0.899	–	–	–	Lin et al. (40)
circANTXR1	0.76	0.668–0.8517	–	–	Huang et al. (42)
circTMEM45A (hsa_circ_0066659)	0.888	0.823–0.954	–	–	Zhang et al. (45)
hsa_circ-0004277	0.816	0.741–0.891	58.3	96.7	Zhu et al. (46)
CircWHSC1	0.8692	–	–	–	Lyu et al. (48)
hsa_circ 0070396^*^	0.8574	0.8025–0.9122	62.16	98.15	Lyu et al. (50)
hsa_circ 0070396 + AFP^*^	0.9384	0.9037–0.9732	81.98	100	Lyu et al. (50)
hsa_circ 0070396^$^	0.7741	0.6955–0.8526	76.58	68	Lyu et al. (50)
hsa_circ 0070396 + AFP^$^	0.8499	0.7893–0.9105	71.17	86	Lyu et al. (50)
hsa_circ 0070396^#^	0.6609	0.5746–0.7472	46.85	81.03	Lyu et al. (50)
hsa_circ 0070396 + AFP^#^	0.7476	0.6719–0.8233	68.47	74.14	Lyu et al. (50)
hsa_circ_0051443	0.8089	–	–	–	Chen et al. (58)
hsa_circ_0028861	0.79	0.72–0.87	67.86	82.69	Wang et al. (12)
hsa_circ_0028861 in AFP (−) HCC	0.78	0.67–0.90	71.43	80.77	Wang et al. (12)
hsa_circ_0028861 in small HCC	0.81	0.73–0.89	70	80.77	Wang et al. (12)
hsa_circ_0028861 in Stage (I–II) HCC	0.82	0.74–0.91	71.43	82.69	Wang et al. (12)
hsa_circ_0028861 +AFP	0.86	0.80–0.93	76.36	86.27	Wang et al. (12)
hsa_circ_0004001	0.79	–	76.19	81.25	Sun et al. (56)
hsa_circ_0004123	0.73	–	66.67	84.38	Sun et al. (56)
hsa_circ_0075792	0.76	–	80.95	68.75	Sun et al. (56)
hsa_circ_0004001 + hsa_circ_0004123 + hsa_circ_0075792	0.89	–	90.5	78.1	Sun et al. (56)

‐, not provided; The AUCs for distinguishing HCC from HD (^*^), CHB (**^$^**), cirrhosis (**^#^**) groups.

**Table 4:** Exosomal circRNAs serve as potential prognostic biomarkers for HCC.

CircRNAs	Prognosis	HR	95 CI%	Ref.
circ-0072088 (low vs. High)	OS	0.475	0.245–0.922	Lin et al. (40)
circUHRF1 (High vs. Low)	OS	1.339	0.944–2.045	Zhang et al. (41)
circUHRF1 (High vs. Low)	PR	1.762	1.172–2.428	Zhang et al. (41)
circRNA-SORE (High vs. Low)	RFS	2.281	1.074–4.845	Xu et al. (44)
circWDR25 (High vs. Low)	OS	1.918	1.252–2.940	Liu et al. (49)
circWDR25 (High vs. Low)	DFS	1.732	1.238–2.423	Liu et al. (49)
circAKT3 (High vs. Low)	OS	1.89	1.04–3.01	Luo et al. (55)
circAKT3 (High vs. Low)	RFS	3.14	1.29–6.21	Luo et al. (55)

OS, Overall Survival; PR, postoperative recurrence; RFS, Progression-Free Survival; DFS, Disease Free Survival.

Hsa_circ_0028861 was downregulated in patients with HCC and might influence HCC progression by regulating its targeted miRNAs and downstream tumor-related signaling pathways (12). Wang et al. (12) showed that hsa_circ_0028861 was identified as a novel diagnostic biomarker for HCC diagnosis with an area under the receiver operating characteristic (ROC) curve (AUC) of 0.79 (95 CI%: 0.72–0.87) for discriminating HCC from chronic HBV and cirrhosis individuals, a sensitivity of 67.86% and a specificity of 82.69%. Moreover, Hsa_circ_0028861 could identify small (AUC = 0.81), early-stage (AUC = 0.82), and AFP-negative (AUC = 0.78) HCC. The combination of hsa_circ_0028861 and AFP exhibited better diagnostic ability (AUC = 0.86, a sensitivity of 76.36% and a specificity of 86.27%). Lyu et al. (50) demonstrated that circ_0070396 was upregulated in plasma-derived exosomes and the combination of hsa_circ_0070396 and AFP displayed higher diagnostic value. The combination has higher diagnostic ability with an ROC of 0.9384 (0.9037–0.9732), 0.8499 (0.7893–0.9105), and 0.7476 (0.6719–0.8233), respectively, for distinguishing HCC from healthy donors, chronic hepatitis B, and cirrhosis groups, than single indicators. Circ_0070396 might be a potential diagnostic biomarker for HCC. Additionally, Sun et al. (56) found that hsa_circ_0004001, hsa_circ_0004123, and hsa_circ_0075792 were upregulated in human blood exosomes from patients with HCC. Sun et al. (56) demonstrated that the diagnostic performance of hsa_circ_0004001, hsa_circ_0004123, and hsa_circ_0075792 exhibited higher sensitivity and specificity. When combined with these three biomarkers, the diagnostic performance was further improved to a sensitivity of 90.5% and an AUC of 0.89. They indicated that the combination of the three circRNAs can be used as a valuable diagnostic biomarker in HCC (56). Moreover, Lin et al. (40) showed that circ-0072088 was mainly secreted by HCC cells *via* exosomes and its expression was significantly higher in HCC tissues and cells than in paracancerous tissue and healthy hepatic cells, and ROC curve analysis showed that circ-0072088 had a high diagnostic value for HCC, with an AUC of 0.899. Some studies discovered that exosome circTMEM45A, circANTXR1, circ-0004277, and circWHSC1 were all upregulated in HCC tissues and cell lines. ROC curves were used to examine their diagnostic values, and they found the AUCs to be 0.888, 0.76, 0.816, and 0.8692, respectively. These results suggest that exosomal circRNAs are diagnostic biomarkers for HCC (42, 45, 46, 48). Furthermore, Chen et al. (58) analyzed the circ-0051443 expression in 60 patients with HCC and 60 healthy subjects, and found that its expression in patients with HCC was significantly lower than that in healthy controls. Circ-0051443 also showed a reliable performance in diagnosing HCC with an AUC of 0.8089.

Liu et al. (49) indicated that high expression of circWDR25 in adjacent tissues was closely related to the poor prognosis of HCC patients after radical hepatectomy. According to Cox regression analyses, circWDR25 was an independent factor for overall survival rate (HR = 1.918; 95% CI: 1.252–2.940, P = 0.003) and tumor free survival (TFS) rate (HR = 1.732; 95% CI: 1.238–2.423, P =0.01) for HCC patients. CircWDR25 has the potential to be used as a screening and monitoring indicator for patients with high recurrence risk HCC. Furthermore, the expression of exosomal circAKT3 in HCC patients was significantly increased compared with healthy subjects. Patients with high exosomal circAKT3 had higher risk of tumor recurrence (HR = 3.14; 95% CI: 1.29–6.21, P = 0.012) and mortality (HR = 1.89; 95% CI, 1.04–3.01), P = 0.048) (55). Additionally, the study by Xu et al. (44) on 60 patients with HCC showed that high circRNA-SORE expression was closely associated with sorafenib resistance. In multivariate Cox regression analysis, the expression level of circRNA-SORE was positively correlated with HCC recurrence-free survival (RFS) (HR = 2.281; 95% CI: 1.074–4.845, P = 0.032) for HCC patients. The study by Zhang et al. (41) found increased levels of exosomal circUHRF1, indicated NK cell dysfunction, resistance to anti-PD1 immunotherapy, and high cumulative recurrence (HR = 1.762; 95% CI: 1.172–2.428, P = 0.019) for HCC patients. Besides, Lin et al. (40) found a notably decreased 5-year survival rate in those with high circ-0072088. The results indicated that high expression of circ-0072088 was closely correlated with an unfavorable prognosis and may be involved in the development of HCC. To reduce the risk of recurrence and improve prognosis, a follow-up of HCC patients with high expression of exosomal circRNAs after surgery is needed.

## Conclusions and Outlooks

HCC exacts a heavy disease burden and is currently the second most common cause of cancer-related deaths worldwide. Most HCC patients are diagnosed at an advanced stage. The risk factors for HCC mainly include chronic hepatitis B, hepatitis C, alcohol addiction, metabolic liver disease, aflatoxins, and aristolochic acid exposure. The therapeutic effect of HCC is poor because of the heterogeneity of the etiology, mutation spectrum, and chemotherapy-resistant nature. Even with complete HCC tumor resection, the carcinogenic tissue microenvironment in the remnant liver can lead to the recurrence of HCC, which progresses to a poor prognosis. Thus, it is necessary to provide specific and early diagnostic biomarkers to detect HCC for more optimal therapies and improved patient prognosis.

CircRNAs are single-stranded RNAs that form covalently closed loops and are stable in exosomes. Exosomes are known as important messengers of the cross-talk between tumor and immune cells. Accumulating studies have demonstrated the promoter or suppressor roles of circRNAs in carcinogenesis, progression, and metastasis of HCC. Exosomal circRNAs have been expressed in HCC tissues, blood, urine, and cell supernatant. The expression dysregulation of exosomal circRNA was tightly related to HCC initiation and progression *via* various mechanisms. Thus exosomal circRNAs can influence HCC cell proliferation, angiogenesis, metastasis, and other biological processes. This review primarily provides that exosomal circRNAs can serve as diagnostic and prognostic biomarkers for HCC and may be potential therapeutic targets for HCC treatment.

The current research results show that whether as diagnostic and prognostic markers or therapeutic monitoring markers of HCC, exosome derived cicrRNAs have a good prospect, but they also have many limitations. First, clinical doctors can detect exosomal circRNAs to diagnose early-stage HCC and monitor the recurrence and metastasis of HCC, but the above study results come from some small sample case–control studies or animal or cell experiments, so the findings may be over interpreted. More multicenter and multi-ethnic large cohorts in the future are needed to validate the actual diagnostic or prognostic effects of exosomal circRNAs. Second, there are still abundant exosomes that have not been discovered whose biological function is unknown. Capturing tissue-specific and disease-specific exosomes and measuring exosomal circRNA profiles may be an important strategy to track the incidence, recurrence, and metastasis of HCC. Further understanding of the molecular mechanisms and downstream signaling pathways of these functional circRNAs that have been found will identify the molecular targets for treatment of HCC. Exosomal circRNAs-based therapies may be introduced for the precise treatment of HCC in the future.

## Author Contributions

ZY and YJ wrote the manuscript. ZY made the figure and tables. All authors listed have made a substantial, direct, and intellectual contribution to the work and approved it for publication.

## Funding

This work was supported by the Natural Science Foundation (LY20H200005 and LY19H200003) of Zhejiang Province.

## Conflict of Interest

The authors declare that the research was conducted in the absence of any commercial or financial relationships that could be construed as a potential conflict of interest.

## Publisher’s Note

All claims expressed in this article are solely those of the authors and do not necessarily represent those of their affiliated organizations, or those of the publisher, the editors and the reviewers. Any product that may be evaluated in this article, or claim that may be made by its manufacturer, is not guaranteed or endorsed by the publisher.
